# On Military and Camp Hospitals, and the Health of Troops in the Field

**Published:** 1862-07

**Authors:** 


On Military and Camp Hospitals, and the Health of Troops in the Field. Being the results of a Commission to inspect the Sanitary Arrangements of the French Army, and incidentally of other armies in the Crimean War. By L. Bandens, Inspector and Member of the Council of Health, of the French Armies, formerly Surgeon-in-Chief, and first Professor of the Perfecting School of Val de Grace, &c., &c.

Translated and Annotated by Franklin B. Hough, M.D., late an Inspector of the U. S. Sanitary Commission. Bailliere Brothers, 440 Broadway, New York.

This is a neatly published volume of 260 small sized octavo pages; and decidedly one of the most useful books that has been thus far called into existence by our present war.

It contains, in a concise and readable form, the results of sanitary measures, both good and bad, adopted by the French and English armies during the Crimean War. It should be carefully read by all who are intending to enter the medical department of our army.

